# Adipose-Derived Stem Cell Treatment Induces Early-Term Hes1 Upregulation in a Sox9- and Notch1-Independent Manner in a Rat Model of Bile Duct Ligation

**DOI:** 10.3390/biomedicines14030657

**Published:** 2026-03-13

**Authors:** Basri Satılmış, Egemen Çiçek, Serdar Karakaş, Koray Kutlutürk, Elif Kayhan, Mehmet Gül, Emrah Otan, Tevfik Tolga Şahin, Sezai Yılmaz

**Affiliations:** 1 Liver Transplant Institute, İnönü University, 44280 Malatya, Turkey; dregemencicek@gmail.com (E.Ç.); serdar.karakas@inonu.edu.tr (S.K.); kkutluturk@gmail.com (K.K.); otanemrah@yahoo.de (E.O.); tevfiktolgaa@gmail.com (T.T.Ş.); sezai.yilmaz@inonu.edu.tr (S.Y.); 2 Department of Surgery, Faculty of Medicine, İnönü University, 44280 Malatya, Turkey; 3 Department of Histology and Embryology, Faculty of Medicine, Malatya Turgut Özal University, 44900 Malatya, Turkey; elif.kayhan@ozal.edu.tr; 4 Department of Histology and Embryology, Faculty of Medicine, İnönü University, 44280 Malatya, Turkey; mehmet.gul@inonu.edu.tr

**Keywords:** adipose-derived stem cell, biliary regeneration, bile duct ligation, Hes1

## Abstract

**Background/Objectives:** Bile duct ligation (BDL), characterized by marked inflammation and fibrosis, effectively mimics many clinical conditions and is a valuable tool for investigating biliary regeneration. Our objective was to clarify the therapeutic benefits of adipose-derived stem cell (ADSC) treatment and signaling pathways mediating regenerative processes in a rat model of BDL. **Methods:** The BDL model was performed on Sprague–Dawley rats, and ADSC was administered intrasplenically at a dose of 10^6^ cells per animal. Liver function tests, gene and protein expression analyses, histological evaluation, and immunohistochemistry staining were performed to assess liver function, signaling pathways, inflammation, and fibrosis. **Results:** ADSC treatment returned liver function to sham levels. ADSC upregulated the Hes1 gene and protein expression in the early and late term. Inflammation, fibrosis, and total damage scores were decreased following ADSC treatment compared with the control. Immunohistochemistry staining revealed higher CD90, CD44, and CD29 stem cell marker expression in the ADSC treatment group. **Conclusions:** ADSC administration reduced fibrosis and biliary damage and restored liver function, potentially in a manner mediated by upregulated Hes1 expression, supporting its promise in biliary regeneration.

## 1. Introduction

There are two types of cholestatic diseases: obstructive jaundice, characterized by mechanical obstruction of the flow of bile into the duodenum, and functional and hepatocellular cholestasis, defined as functional problems that prevent the normal production of bile by hepatocytes [1]. These clinical conditions include extrahepatic bile duct injuries from laparoscopic cholecystectomy, primary biliary cirrhosis (which involves complete damage to intrahepatic bile ducts due to cholestasis), and cholangiocarcinomas and biliary tumors (which initially appear as an obstruction in the extrahepatic bile duct) [2,3,4]. Cholestasis triggers inflammation and fibrosis in the liver parenchyma [5]. In cholangiopathies, cytokines and growth factors are released as a result of severe inflammation and promote cholangiocyte proliferation, diffuse fibrosis, and apoptosis [6]. The bile duct ligation (BDL) model, where inflammation and fibrosis develop intensely, simulates many clinical conditions and is ideal for studying hepatic and biliary regeneration [7]. BDL induces cholangiocyte proliferation, triggers stellate cell activation, and promotes fibrosis [8,9].

Mesenchymal stem cells (MSCs) are a promising cellular treatment option for various clinical conditions due to their ability to transform into various tissue types, self-renewal properties, and infinite proliferation capacity [10,11,12,13]. Adipose-derived MSCs (ADSC) are gaining increasing interest in regenerative medicine because they can form more colonies and have greater in vitro proliferative potential compared with MSCs from different sources [12]. In experimental models, the immunomodulatory properties of ADSC and their capacity to differentiate into liver parenchymal cells make ADSC a valuable therapeutic option [14,15].

Hepatic and biliary regeneration are not separate phenomena and involve almost the same stages [16,17]. Following damage, hepatocytes and cholangiocytes become less specialized and more alike, which allows them to differentiate from one another [18]. Cholangiocytes proliferate and initiate a cellular response known as ductular reaction (DR) to compensate for cell loss during chronic bile duct injury [19]. The specific type of injury results in a reprogramming process that determines the ultimate destination of the DR. Noninvasive DR is primarily found in the periportal area, whereas invasive DRs are marked by ductular proliferation that spreads into the lobular parenchyma [19]. The mechanism behind the noninvasive DR in a BDL model of liver injury and fibrosis was found to be primarily the proliferation of large cholangiocytes, which are found in larger bile ducts and are more prone to injury [19,20]. This type of DR in acute bile duct obstruction is driven by the proliferation of previously formed cholangiocytes and leads to the formation of widened biliary tubes [21]. When cholangiocytes are damaged, they activate intracellular signaling mechanisms for maintaining cell communication and triggering DR [22]. The differences in signaling pathways and gene expression levels in cholangiocytes have been observed [19]. The proliferation of cholangiocytes is affected by various signaling pathways such as the Hippo pathway, Wnt/β-catenin, Notch, and Hedgehog, which play a role in the development of DR [23,24,25,26].

Although ADSC can regulate different liver cells in the regeneration process, the role of ADSC as a modulator of the regeneration signaling pathway has not yet been explored. This study’s main aim was to profile the effects of ADSC on signaling pathways involved in cholangiocyte proliferation in the BDL model.

## 2. Materials and Methods

### 2.1. Donor Animal Procedures and Isolation of ADSC

All stages of the experimental study were performed per the European Union’s Directive 2010/63/EU and were approved by the Animal Experiments Ethics Committee. The rats were provided from the Experimental Animal Research Facility of İnönü University and kept in climate-controlled chambers with a humidity level of 60% and a 12:12 h light cycle. Food and tap water were provided ad libitum. Female Sprague–Dawley rats, 5–6 months old, were used as donor animals. The animals were anesthetized with xylazine (5 mg/kg) and ketamine (80 mg/kg). We used a standard technique for excision of the inguinal fat pads and isolation of the ADSC as described previously [13,27,28]. Briefly, the inguinal and abdominal regions were shaved and sterilized with povidone iodine. Bilateral inguinal and midline skin incisions were performed, and the skin flaps were advanced to the flank region. The inguinal and flank fat pad was excised and immersed in chilled phosphate-buffered saline (Wisent Bioproducts, Saint-Jean-Baptiste, QC, Canada, 311-010-CL), penicillin–streptomycin–neomycin (Sigma, St. Louis, MO, USA, P4083), and amphotericin B (Biological Industries, Kibbutz Beit Haemek, Israel, 03-028-1B). The adipose tissues were rapidly transferred to the Inonu University Liver Transplant Institute Hepatology Research Laboratories.

All procedures were performed in biological safety cabinets (Nüve, Ankara, Turkey, MN120). Once the adipose tissues were transferred to our research laboratory, they were immersed in phosphate-buffered saline sequentially in 6-well plates (SPL, Pocheon, Republic of Korea, 30006) for irrigation and removal of the macroscopic blood components. The tissues were transferred to a 100 mm Petri dish (Orange Scientific, Braine-l’Alleud, Belgium, 4450300) and minced into 1 mm^3^ small pieces until the entire tissues were mashed. The mash was then transferred to a 9 mL cell medium containing high-glucose DMEM (Sigma, D6429) and 10% *v*/*v* penicillin–streptomycin. Next, 1 mL of 0.05% Type II Collagenase was added on top and incubated in a warm water bath (Memmert, Schwabach, Germany, WNB15) at 37 °C for 60 min. During this period, the tubes were vortexed every 5 min for agitation and digestion of the tissues. At the end of the period, trypsin (Sigma, T3924) was added to the solution and incubated for 5 min at 37 °C, and the tissues were agitated via vortex spin. The digestion procedure was stopped by adding fetal bovine serum (FBS) (Sigma, F7524) containing DMEM high-glucose medium, and the tissues were dispersed in the solution. The tissues were centrifuged (Eppendorf, Hamburg, Germany, 5810R) at 4000 rpm and 4 °C for 25 min. The supernatant was discarded, and the pellet was resuspended in cell medium and filtered through 100 μm and 40 μm cell strainers (Labsolute 7696769 and Labsolute 7696767, Bochum, Germany) sequentially. The cell suspension was centrifuged again at 4 °C at 1200 rpm for 5 min. The supernatant was discarded, and the cell pellet was resuspended in null high-glucose DMEM. The cells were counted using Trypan Blue (Biological Industries, 03-102-1B) by mixing equal volumes of cells and Trypan Blue (10 µL each). The cells were loaded on a cell-counting slide and counted with an automated cell counter device (Logos Luna II, Seoul, Republic of Korea), and the live cell count per ml was calculated. The cells were then cultured in a T75 cell culture flask containing high-glucose DMEM, penicillin–streptomycin–neomycin, and amphotericin B. The cells were then incubated at 5% CO_2_ and 37 °C in an incubator (Nüve, EC160). The progression of cell culture and the cell density were evaluated each day using an inverted microscope (Leica, Wetzlar, Germany, DMi8).

### 2.2. The Experimental Model

We used a standard technique to establish the BDL model [7]. Briefly, we used 5–6-month-old 200–250 g female Sprague–Dawley rats as model animals. The animals were anesthetized with xylazine (5 mg/kg) and ketamine (80 mg/kg). The anterior abdominal wall of the animals was shaved and sterilized with povidone iodide, and a midline incision was performed. The stomach and duodenum were retracted caudally, and the common channel was exposed. A small vascular clip (Horizon, Teflex Co., Research Triangle Park, NC, USA) was applied to the common channel near the duodenum to avoid any vascular injuries.

The animals were divided into 3 groups with 12 animals in each. The first group was the sham group; no procedures were performed on these animals. They were sacrificed, and blood samples and liver tissues were collected. The second group was the control group, wherein common bile duct ligation was performed, and 100 µL of phosphate-buffered saline (PBS) (Wisent Bioproducts, 311-010-CL) was injected into the spleens of the animals. The third group was the ADSC-treated group, wherein we performed bile duct ligation, and each animal received an injection of 10^6^ ADSCs/100 µL of PBS intrasplenically. The splenic injection was chosen to simulate the intraportal injection of the ADSC. As this is a well-known method for the formation of hepatic metastasis of experimental tumor models, we used it as a mode of access to treatment in our experiments [29].

We initially aimed to use 6 animals per study group (except the sham-operated group). However, after performing the BDL model, we had 7 mortalities in total. Therefore, the POD5 control group was reduced to 2 animals, and the POD5 ADSC groups were reduced to 3 animals. We had no mortality in the other groups. Evaluating POD15 would be more informative regarding the overall effects of ADSC treatment. The losses in the early term were more tolerable.

### 2.3. Differentiation of ADSC into Adipocytes, Osteocytes, and Chondrocytes

The adipocyte differentiation of ADSC was assessed with the StemPro Adipogenesis Differentiation Kit (Gibco, Grand Island, NY, USA, A10070-01). Cells were seeded at a density of 10^4^ cells/cm^2^/well and incubated overnight at 37 °C with 5% CO_2_. The cell culture medium was replaced with adipogenesis differentiation medium for 14 days of incubation. The cells were fixed with 4% formaldehyde, stained with Oil Red O, and imaged.

Osteocyte differentiation of ADSC was assessed with the StemPro Osteogenesis Differentiation Kit (Gibco, A10072-01). Cells were seeded at a density of 5 × 10^3^ cells/cm^2^/well and incubated overnight at 37 °C with 5% CO_2_. The cell culture medium was replaced with osteogenesis differentiation medium for 21 days of incubation. The cells were fixed with 4% formaldehyde, stained with 2% Alizarin Red, and imaged.

Chondrocyte differentiation of ADSC was assessed with the StemPro Chondrogenesis Differentiation Kit (Gibco, A10071-01). Micromass cultures were generated by seeding 5 µL droplets of 10^7^ viable cells/mL and incubated overnight at 37 °C with 5% CO_2_. The cell culture medium was replaced with chondrogenesis differentiation medium for 14 days of incubation. The cells were fixed with 4% formaldehyde, stained with 1% Alcian Blue, and imaged.

### 2.4. Blood Analysis

Blood samples were centrifuged at 2000× *g* at 4 °C for 10 min, and the sera were kept at −86 °C in a freezer (Panasonic, Kadoma, Japan, MDF-DU502VX-PE). AST, ALT, ALP, and GGT levels were determined using ELISA kits (Andygene, Beijing, China, AD2027Ra, AD2041Ra, AD2042Ra, and AD2231Ra), and bilirubin levels were determined with an assay kit (BioVision, Milpitas, CA, USA, K553-100), according to the manufacturer’s instructions. Measurements were performed with a microplate reader (Biotek, Synergy H1m, Winooski, VT, USA).

### 2.5. RNA Extraction and RT-PCR

Total RNA was isolated from liver tissue using a kit (Qiagen, RNeasy Mini Kit), quantified, and reverse-transcribed to cDNA (Qiagen, RT^2^ HT First Strand Kit). Amplification was performed via real-time PCR (Qiagen, Rotor-Gene Q) using the RT^2^ SYBR Green qPCR Master Mix, and specific primers for β-catenin (Cat No.: PPR57457A; RefSeq Accession No.: NM_053357.2), Yap1 (Cat No.: PPR54845A; RefSeq Accession No.: NM_001034002.2), Notch1 (Cat No.: PPR47971A; RefSeq Accession No.: NM_001105721.1), Gli1 (Cat No.: PPR53356A; RefSeq Accession No.: NM_001191910.1), Sox9 (Cat No.: PPR53329A; RefSeq Accession No.: NM_001081628.5), Hes1 (Cat No.: PPR46895C; RefSeq Accession No.: NM_024360.3), and GAPDH (Cat No.: PPR06557B; RefSeq Accession No.: NM_017008.4) were obtained from Qiagen, Hilden, Germany.

### 2.6. Western Blotting

Liver samples were lysed with ice-cold RIPA buffer (Sigma, R0278) containing a 1% protease and phosphatase inhibitor cocktail (Sigma, PPC1010). The proteins were loaded onto a 4–20% gel and transferred onto a PVDF membrane. The membranes were probed with the primary antibodies β-catenin (Cell Signaling, Danvers, MA, USA, 8480), YAP/TAZ (Cell Signaling, 8418), Notch1 (Cell Signaling, 3608), Gli1 (Cell Signaling, 3538), Sox9 (Cell Signaling, 82630), Hes1 (Cell Signaling, 11988), and β-actin (Cell Signaling, 4970) and a secondary antibody (Cell Signaling, 7404). Bands were visualized using a Clarity Max Western ECL Substrate (Bio-Rad, Hercules, CA, USA, 1705062) and a gel imaging system (Bio-Rad, ChemiDoc), and band quantification was performed using the Bio-Rad Image Lab software Version 6.1.0 build 7.

### 2.7. Histology and Immunohistochemistry

Liver samples were fixed in formalin, embedded in paraffin, cut into 6 µm sections, and mounted on slides. The sections were stained with hematoxylin–eosin (H&E) and incubated for immunohistochemical (IHC) analysis with CD90, CD45, CD29, and CD44 antibodies (Santa Cruz Biotechnology, Santa Cruz, CA, USA) diluted at 1:100. H&E and IHC-stained liver sections were examined using a Nikon Eclipse Ni-U light microscope (Tokyo, Japan), a Nikon DS-Fi3 camera, and the Nikon NIS-Elements Documentation 5.02 image analysis program. The H&E-stained sections were examined by histologists blinded to the experimental groups. Liver injury was scored using a system from 0 to 3 (0, normal; 1, mild; 2, moderate; 3, severe) for necrosis, fibrosis, and inflammatory cell infiltration. The maximum total injury was set as 9 [30].

DR was graded as absent (0, 1–2 bile duct profiles per portal tract), mild (1, 3–5 bile duct profiles per portal tract), moderate (2, 6–10 bile duct profiles per tract), or marked (>10 bile duct profiles per tract) [31]. Immunostaining for antibodies was performed according to the BOND-MAX Fully Automated IHC Staining System protocol (Leica-microsystems, Buffalo Grove, IL, USA). This semi-quantitative method calculates the product of the percentage of positively stained cells and the weighted intensity of the staining: H Score = Ʃ Pi (i + 1), where Pi is the percentage of stained cells (0–100%) in each intensity category. The intensity of staining (i) was characterized as absent (0), weak but detectable above the control (1), distinct (2), or very strong (3).

### 2.8. Statistical Analysis

The Shapiro–Wilk test was used to assess the normal distribution of continuous variables. Since none of the continuous variables were not distributing normally expressed all our continuous variables were expressed as the median and interquartile range. The Kruskal–Wallis ANOVA test was used to compare continuous variables across multiple groups. The Mann–Whitney U test was used to compare continuous variables between two groups. A *p*-value of less than 0.05 was considered statistically significant. All statistical analyses were performed using SPSS version 27 (IBM, Armonk, NY, USA).

## 3. Results

### 3.1. Differentiation of Adipocytes into Cells from Different Tissue Types

**Figure 1:**
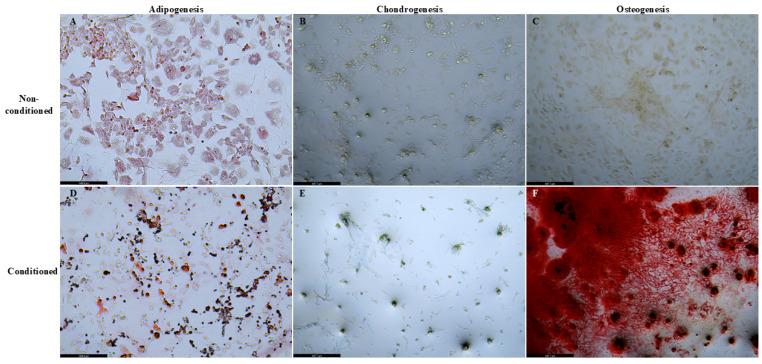
Summary of the multilineage differentiation capacity of the ADSC. (**A**–**C**) show the non-conditioned control group showing slight adipogenic differentiation (**A**) and no chondrogenic or osteogenic differentiation (**B**,**C**) in the medium. Meanwhile, conditioned ADSC showed differentiation into all lineages, including adipogenic (**D**), chondrogenic (**E**), and osteogenic (**F**) differentiation. Successful differentiation was confirmed using specific staining: Oil Red O for adipocytes (magnification, 200×; scale bar length, 250 µm), Alizarin Red for osteocytes, and Alcian Blue for chondrocytes (magnification, 100×; scale bar length, 500 µm).

We observed that the isolated ADSC differentiated into adipocytes, chondrocytes, and osteocytes using different in vitro differentiation protocols. Our results are summarized in Figure 1.

### 3.2. Biochemical Analyses of the Liver Function Tests

**Figure 2:**
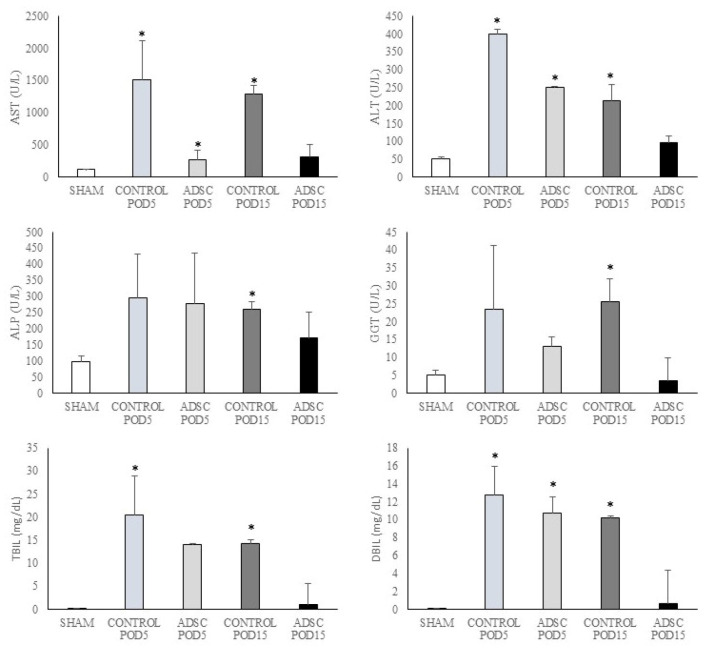
The temporal changes in the liver function tests, including AST, ALT, ALP, GGT, total bilirubin, and direct bilirubin levels, in the study groups. All results are expressed as medians ± IQR, and a significance level of *p* < 0.05 was adopted for the analysis. * versus sham group.

The results of the biochemical analyses for the study groups are summarized in Figure 2. In the POD15 ADSC group, the levels of AST, ALT, ALP, GGT, total bilirubin, and direct bilirubin were observed to return to near-sham group levels. The difference between the sham group and the POD15 ADSC group was not statistically significant (*p* > 0.05). Notably, AST levels in the ADSC-treated group decreased to sham group levels as early as post-operative day 5 (POD5). In contrast, the POD15 levels of these parameters in the control group were found to be significantly higher than those in the sham group (AST: *p* = 0.001, ALT: *p* = 0.001, ALP: *p* = 0.002, GGT: *p* = 0.001, total bilirubin: *p* = 0.001, and direct bilirubin: *p* = 0.001). All data are summarized in Supplementary Tables S1 and S2.

### 3.3. The Results of Gene Expression Analyses

**Figure 3:**
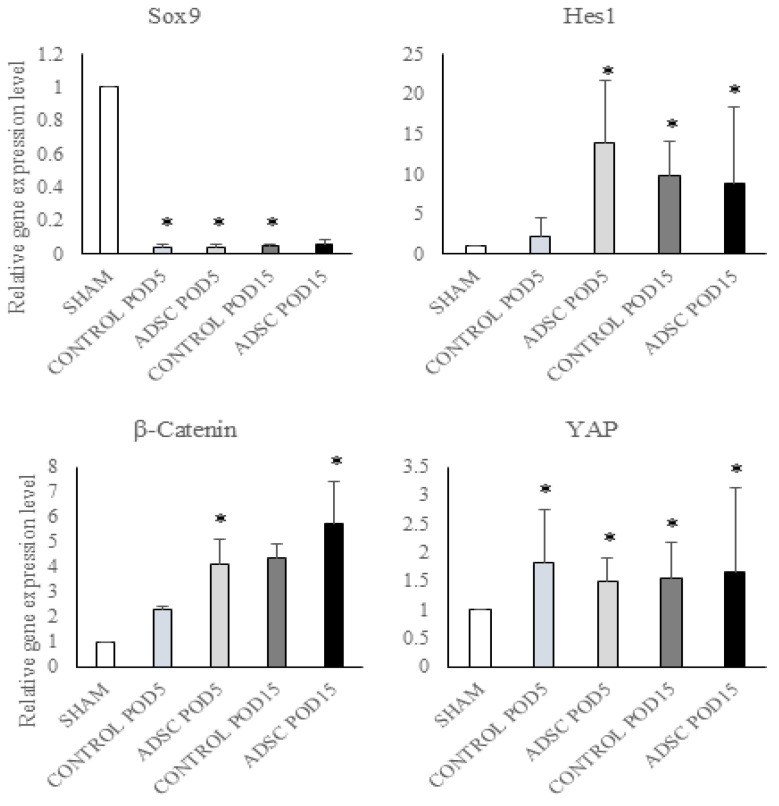
The effect of ADSC treatment on regeneration pathways at the gene level was determined using RT-PCR. Changes in gene expression levels were normalized using GAPDH as a housekeeping gene. Any *p*-value of less than 0.05 was considered statistically significant. * versus sham.

Sox9 exhibited lower gene expression levels in all control and ADSC groups compared with the sham group, independent of the application and duration (POD5 control vs. sham: *p* = 0.002; POD5 ADSC vs. sham: *p* = 0.005; POD15 control vs. sham: *p* = 0.003). Hes1 expression was higher in the early period in the POD5 ADSC group compared with the sham group (POD5 ADSC vs. sham: *p* = 0.001) and in the late period in the POD15 control and POD15 ADSC groups compared with the sham group (POD15 control vs. sham: *p* = 0.027; POD15 ADSC vs. sham: *p* = 0.002). β-catenin expression was higher in the ADSC-treated groups in both periods compared with sham group (POD5 ADSC vs. sham: *p* = 0.018; POD15 ADSC vs. sham: *p* = 0.001). YAP expression was higher in the control and ADSC groups compared with the sham group (POD5 control vs. sham: *p* = 0.027; POD5 ADSC vs. sham: *p* = 0.037; POD15 control vs. sham: *p* = 0.009; and POD15 ADSC vs. sham: *p* = 0.014). The gene expression results for the study groups are summarized in Figure 3.

### 3.4. The Results of Protein Expression Analyses

**Figure 4:**
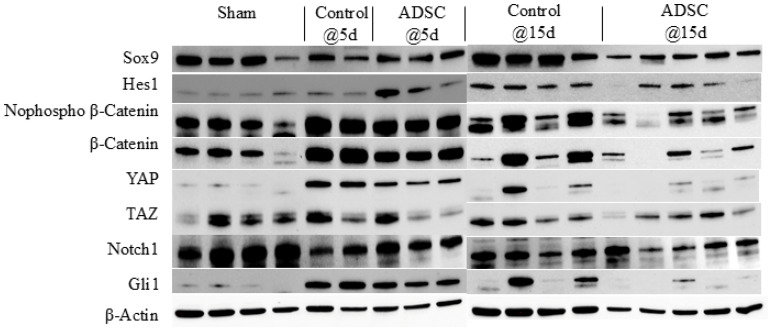
The effect of ADSC treatment on the temporal expression levels of proteins in the regeneration pathways was determined using Western blotting. The procedure was repeated with 4 samples for the sham group, 2 for the POD5 control group, 3 for the POD5 ADSC group, 4 for the POD15 control group, and 5 for the POD15 ADSC group.

**Figure 5:**
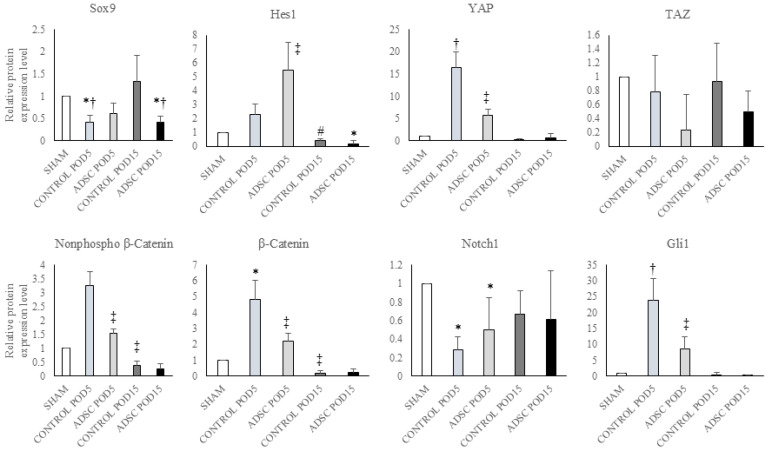
The relative protein expression changes in the regeneration pathways following ADSC treatment were normalized using β-actin as a housekeeping protein. Any *p*-value < 0.05 was considered statistically significant. * versus sham, # versus POD5 control, † versus POD15 control, and ‡ versus POD15 ADSC.

The results of the protein expression analysis are summarized in Figure 4. Sox9 protein expression levels were reduced by half in both the POD5 and POD15 ADSC groups compared with the sham group (POD5 ADSC vs. sham: *p* = 0.043; POD15 ADSC vs. sham: *p* = 0.002). In the early post-operative period, Hes1 protein expression was higher in the POD5 ADSC group compared with the POD15 ADSC group (*p* = 0.001). Expression levels of non-phospho-β-catenin and β-catenin were higher in the POD5 control and POD5 ADSC groups compared with the sham group. β-catenin expression in the POD5 control group significantly increased compared with the sham group (*p* = 0.05). In contrast with these early findings, the levels of these proteins in both the POD15 control and POD15 ADSC groups decreased more than in the sham group (POD15 ADSC vs. POD5 ADSC: *p* = 0.001). YAP levels were higher in the POD5 control and POD5 ADSC groups compared with the sham group, but subsequently decreased in the POD15 control and POD15 ADSC groups to levels below those observed in the early period (POD15 control vs. POD5 control: *p* = 0.05; POD15 ADSC vs. POD5 ADSC: *p* = 0.001). While TAZ protein expression remained relatively unchanged in the control groups, its levels were lower in both the POD5 and POD15 ADSC groups compared with the sham group. The expression of both YAP and TAZ proteins was suppressed in the POD15 ADSC group. Finally, Gli1 expression was suppressed by approximately 50% in both the POD5 and POD15 ADSC groups compared with their respective control groups (POD15 control vs. POD5 control: *p* = 0.032; POD15 ADSC vs. POD5 ADSC: *p* = 0.001). The relative changes in protein expression levels for all study groups compared with the sham group are summarized in Figure 5.

### 3.5. Histopathological Analysis

#### 3.5.1. Macroscopic Examination

**Figure 6:**
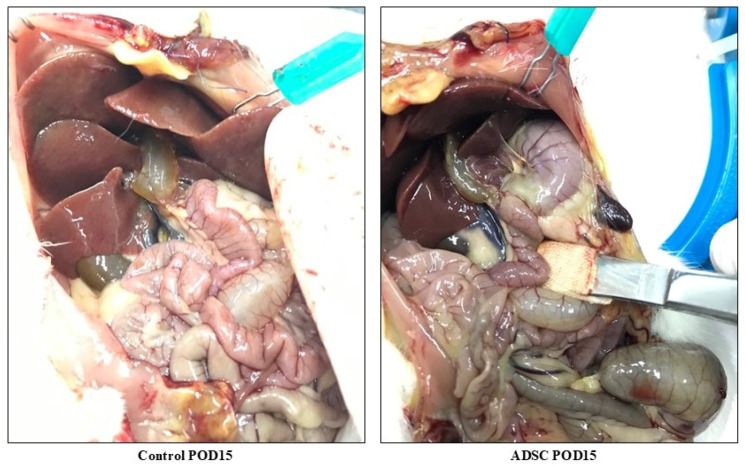
Macroscopic view of the liver and bile ducts on POD15 in animals from the control and ADSC groups following common bile duct ligation.

The livers in the control (untreated) group had a markedly steatotic appearance, were firm to the touch, and exhibited severe bile duct dilatation. In contrast, the livers in the ADSC-treated group showed less bile duct dilatation and more closely resembled a normal anatomical structure (Figure 6).

#### 3.5.2. Histological Hematoxylin–Eosin Staining

**Figure 7:**
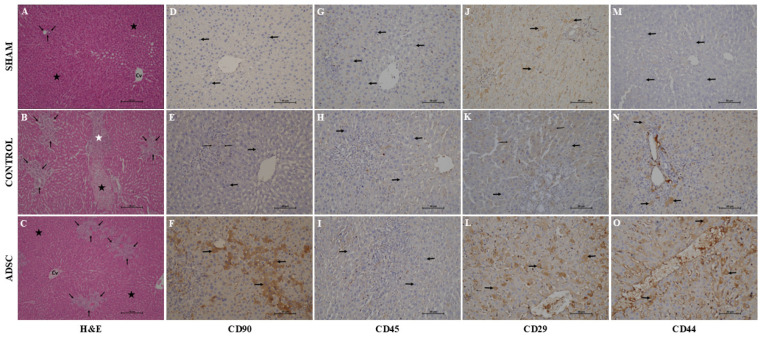
Representative images of H&E staining and immunohistochemistry for CD90, CD45, CD29, and CD44 in liver tissues from POD15 control and ADSC-treated groups, as well as the sham group. Figure (**A**–**C**) summarize the H&E staining in the study groups. (**A**) The normal liver architecture is seen in the sham group. (**B**) Shows the fibrotic bands in the control group (black star represents hepatocyte necrosis, white star represents fibrosis and arrow represents inflammatory cell infiltration in the portal area). (**C**) The fibrotic areas are reduced in the ADSC-treated group (magnification, 100×; scale bar length, 100 µm). Figure (**D**–**F**) summarize the distribution of CD90 (one of the mesenchymal stem cell markers) throughout the hepatic lobule in the study groups (magnification, 200×; scale bar length, 50 µm). (**D**,**E**) show no staining in the sham and control groups, whereas (**F**) shows extensive CD90-positive cells distributed throughout the hepatic lobules. Figure (**G**–**I**) summarize the distribution of CD45 throughout the liver tissue in the study groups (magnification, 200×; scale bar length, 50 µm). Since it is a negative marker, there is no specific staining in any group. Figure (**J**–**L**) summarize the distribution of CD29 throughout the liver tissues in the study groups (magnification, 200×; scale bar length, 50 µm). There is no specific staining in the sham (**J**) and control groups (**K**). Since CD29 is a specific mesenchymal stem cell marker, it is extensively stained in the ADSC-treated group (**L**). Figure (**M**–**O**) summarize the distribution of CD44 throughout the liver tissue in the study groups (magnification, 200×; scale bar length, 50 µm). While there was no specific staining in the sham group (**M**), the control group showed mild to moderate CD44-positive cells throughout the hepatic lobule, which is suggestive of the facultative stem cells located in the Canals of Herring near the portal triads. However, CD44-positive cells were present extensively throughout the hepatic lobule in the ADSC-treated group.

As expected, hematoxylin–eosin staining revealed a normal liver architecture in the sham group (Figure 7). Control group liver samples exhibited significant pathological changes, including parenchymal necrosis, inflammatory cell infiltration, fibrosis, bile duct proliferation within the portal triads, and bridging fibrosis. In the ADSC-treated group, these histopathological changes were markedly resolved.

**Figure 8:**
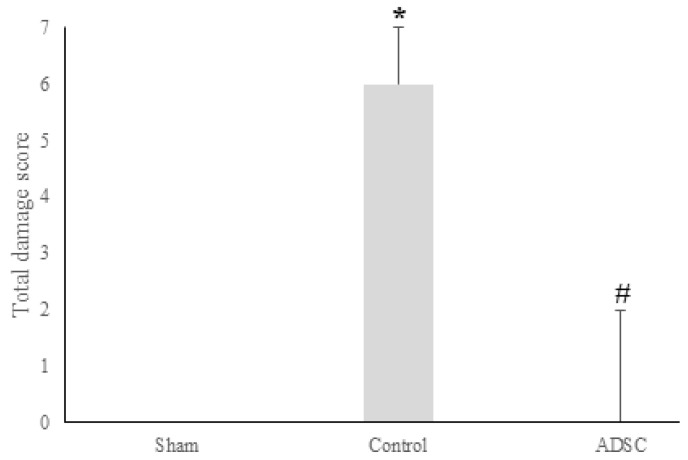
Total damage score, calculated based on necrosis, fibrosis, inflammatory cell infiltration, and ductal proliferation, in liver samples collected on POD15 from the sham, control, and ADSC groups subjected to the common bile duct ligation model. The level of statistical significance was set at *p* < 0.05. * versus sham; # versus control.

**Figure 9:**
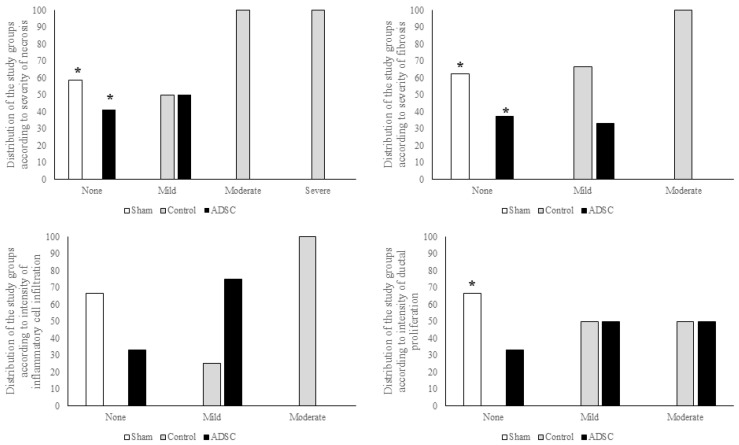
A comparison of the distribution of necrosis, fibrosis, inflammatory cell infiltration, and ductal proliferation in liver samples taken on POD15 from the sham, control, and ADSC groups in the common bile duct ligation model. The level of statistical significance was set at *p* < 0.05. * versus control.

Analysis of fibrosis, necrosis, and DR revealed significant differences among the study groups. All cases showing no DR belonged to the sham group (*p* < 0.001), while cases that did not develop fibrosis or necrosis were from the sham and ADSC-treated groups (*p* < 0.001). However, there was no statistically significant difference among the groups regarding the severity of inflammatory cell infiltration. A summary of the total damage scores for each group is presented in Figure 8. The individual scores for fibrosis, necrosis, and inflammatory cell infiltration, along with inter-group comparisons, are summarized in Figure 9.

#### 3.5.3. Immunohistochemical Examination

**Figure 10:**
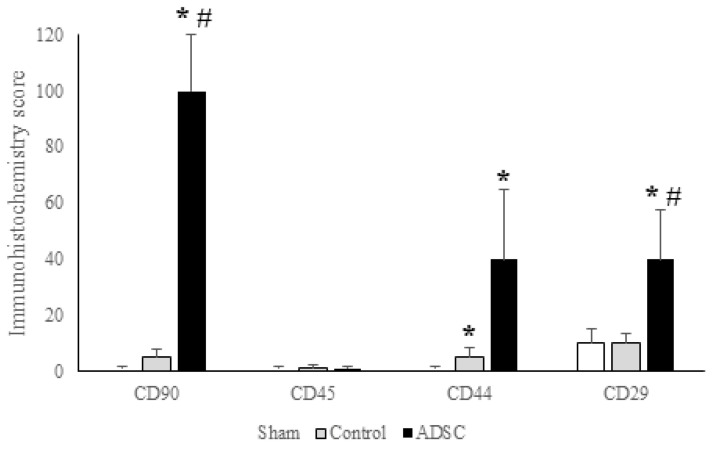
Quantification of immunohistochemistry staining for CD90, CD45, CD29, and CD44 in liver samples taken on POD15 from the sham, control, and ADSC groups in the common bile duct ligation model. Any *p*-value < 0.05 was considered statistically significant. * versus sham; # versus control.

In this study, CD44, CD29, and CD90 were selected as stem cell markers, with CD45 used as a negative marker. The expression scores for CD29 were 10, 10, and 40 for the sham, control, and ADSC groups, respectively. Expression in the ADSC group was significantly higher than in the sham and control groups (*p* < 0.05). Similarly, the CD90 expression score in the ADSC group (100) was significantly higher than that in the control (5) and sham (1) groups (*p* < 0.05). The CD44 expression scores were 1, 5, and 40 for the sham, control, and ADSC groups, respectively. CD44 expression was significantly higher in the ADSC group compared with the other groups (*p* < 0.05). Furthermore, the intensity of CD44 expression in the control group was significantly higher than in the sham group (*p* = 0.035). As expected, CD45 was not expressed in any of the groups. A summary of the immunohistochemical staining intensities across the groups is presented in Figure 10.

## 4. Discussion

This preclinical study examined the effects of ADSC on liver function and the ultrastructural features of biliary channels in a BDL model. Our research offers important insights into how mesenchymal stem cell therapy promotes hepatocellular and cholangiocyte regeneration, which is its key contribution to the literature. While studies using BDL models often focus on preventing or reducing fibrosis, our model showed fibrosis reversal and the restoration of nearly normal liver anatomy, thanks to the regenerative, proliferative, angiogenic, and anti-inflammatory effects of ADSC [14,15,27,32,33,34,35,36,37,38,39]. Our primary finding was that ADSC supported cholangiocyte regeneration and preserved the ultrastructural integrity by altering the expression of Hes1.

Our study demonstrated that ADSC therapy supported the liver’s ultrastructural features and functions. The mesenchymal stem cell markers CD29 and CD90 were found scattered throughout the hepatic lobule, indicating that the intrasplenically injected ADSC distributed throughout the liver. In typical hepatic regeneration, a wave of hepatocyte proliferation moves from the portal triad to the central vein or vice versa [16,40]. Alternative pathways involve progenitor cells at the Canals of Hering, which proliferate and disperse from the portal triad into the hepatic lobule [16,40]. Our results showed that ADSC-mediated regeneration of hepatocytes and cholangiocytes does not display any polarization within the lobule. Additionally, the untreated control group exhibited CD44-expressing cells, indicating that oval cells at the Canals of Hering expressed common stem cell markers [41]. The mechanisms behind cholangiocyte restoration in hepatic regeneration have also not been thoroughly examined. Our study offers valuable insights aimed at filling these significant gaps in the literature. We showed that exogenously administered ADSC spread to all parts of the hepatic lobule. Hara et al. [14] reported that bile duct strictures and cholangitis attacks developed in the first 15–20 days in a biliary anastomosis model in pigs. BDL models created in rodent models showed a similar timeline [7]. Our study’s findings support the literature [14,15,33,34] and revealed that hepatic fibrosis was reduced in the group treated with ADSC.

Sox9 plays a crucial role in the development of the liver, intrahepatic bile ducts (IHBDs), and pancreatic ducts. Alongside Sox4, it is essential for the regulation and maintenance of normal IHBD formation, and IHBD cells accordingly express both proteins [42]. During development, Sox9 and Sox4 engage in a positive feedback loop with Notch1 and Notch2 [43]. The subsequent interaction between Sox9 and the Notch pathway induces Hes1 expression at later stages [44]. Our study found that treatment with ADSC reduces Sox9 expression at both the transcriptional and translational levels. In contrast, we observed an early induction of Hes1 expression, which also increased at both the transcriptional and translational levels. Ductal proliferation is a common reaction to cholestatic liver diseases. The underlying mechanisms involve the proliferation of pre-existing cholangiocytes, the extension of bile ducts to the site of injury, and the biliary metaplasia of periportal hepatocytes [45,46,47,48,49]. This process can be viewed as a regenerative effort in which hepatocytes or oval cells differentiate toward a biliary cell lineage. The transcription factor Sox9 plays a crucial role in ductal proliferation. Preclinical studies using rodent models of BDL have shown that Sox9-positive (Sox9+) hepatocytes appear in the first week, just before ductal proliferation begins [50]. Furthermore, Yoshii et al. [50] reported that the presence of Sox9+ hepatocytes adjacent to portal areas in patients with biliary atresia was associated with a better prognosis compared with patients without them. Therefore, Sox9 expression is indicative of biliary metaplasia of hepatocytes in the periportal area. In our study, although the overall damage scores were lower in the ADSC-treated group, ductal proliferation was comparable between the ADSC-treated group and the untreated control group. Our Western blot analyses revealed that while the ADSC-treated group had higher Sox9 expression than the untreated control group in the early period, this difference did not reach statistical significance. Moreover, in the later phase, the control group had significantly upregulated Sox9 expression, while its expression in the ADSC-treated group was decreasing. The sham group exhibited the highest Sox9 expression; all other groups had significantly lower expression levels in comparison. We believe this is a normal turnover process seen in rodent livers because, in rodents, the expression of Sox4 and Sox9 is typically elevated during the embryonic period to facilitate the development of intrahepatic bile ducts [51,52]. Later in life, its expression is reduced but not entirely lost, and can increase in response to injury or epithelial cell turnover in the bile ducts [52,53]. Sox9-expressing cells are abundant in the normal liver [49]; for example, Sox9+ hepatocytes supply hepatocytes and intrahepatic biliary epithelial cells in normal liver homeostasis [54,55]. Our results support the expression pattern of Sox9 in the normal liver and suggest that the initial response to injury involves reduced Sox9 expression, followed by a gradual recovery during the second week—a pattern observed in the control group. In the ADSC-treated group, however, the elevation in Sox9 (although not statistically significant) began earlier. We propose that sampling at earlier time points or conducting closer surveillance during the first week after BDL could have captured this early surge more definitively. The initial drop in Sox9 expression may suggest that the regenerative process prioritizes replacing lost hepatocyte populations in the early period. This aligns with reports of hepatocyte apoptosis occurring as early as 3 to 7 days following BDL in rodents [53,54,55,56]. Consequently, the earlier increase in Sox9 expression observed in the ADSC-treated group may indicate that ADSC accelerate this regenerative process. This is further supported by the reduced inflammatory damage with ADSC treatment [57]. In our experiment, inflammation was significantly reduced in the ADSC-treated group to levels comparable to the sham group, whereas it was highest in the untreated control group. It should be noted that we did not examine HNF4α in our study, and we could have also assessed the level of hepatocyte differentiation.

The Notch1/Hes1 signaling pathway is known to be upregulated in chronic fibrotic biliary obstructions [58]. Conversely, Hes1 also appears to regulate cholangiocyte proliferation in diseases such as primary sclerosing cholangitis [59]. A study by Marzioni et al. [59] demonstrated that Hes1 was downregulated in patients with primary sclerosing cholangitis, a finding associated with extensive DR. Matsumori et al. [60] showed that Notch1 upregulation increases Hes1 expression to induce cholangiocyte proliferation in preclinical models. We observed a significant increase in Hes1 protein expression, which peaked on POD5 in the ADSC-treated group. This surge in the Hes1 protein was statistically significant and correlated with a simultaneous increase in its gene transcription. Since a similar response was absent in the untreated control group, our findings suggest that ADSC treatment induces Hes1 expression. Hes1 is a key effector of the Notch signaling pathway in IHBD development [61]. Similarly, we observed increased expression of Notch1 in the ADSC-treated group. We noted that protein expression levels varied from the gene expression levels; however, such discrepancies are not uncommon, as mRNA expression may differ from its protein counterpart. In some instances, RT-PCR can explain only 53% of the observed protein expression patterns [62].

YAP and TAZ are closely related paralogs that function cooperatively in both biliary duct development and injury-induced ductular proliferation [63]. Furthermore, both are closely linked to the Sox9/Notch/Hes1 pathway during the development and differentiation of biliary ducts [52]. In our study, YAP and TAZ expression levels in the ADSC-treated group were lower than in the other experimental groups. These findings suggest that ADSC treatment does not have a significant effect on the YAP/TAZ pathway. Although β-catenin is considered dispensable for bile duct generation and biliary epithelial proliferation [64], it is associated with bile duct development and the ductular reaction observed in cholestatic liver disease, where it is closely linked to the Wnt pathway [65]. In our study, β-catenin protein was upregulated during the early period of cholestasis, and this response was more prominent in the untreated control group. In this group, the gene expression of β-catenin was also more persistently increased in both the early and late periods. While the β-catenin gene expression was higher in the ADSC-treated group, this did not translate into its protein expression levels. Nevertheless, both the ADSC-treated and control groups exhibited higher β-catenin protein expression compared with the sham group. The Hippo signaling pathway also plays an important role in biliary development in a Notch-dependent manner [66]. We did not analyze the Hippo pathway in our study; however, this was not relevant to the possible mechanisms of biliary regeneration observed in our study. Both β-catenin and Notch-1 were reduced in the study groups compared with the sham-operated group. For these reasons, the biliary regeneration that we observed in our study is independent of these pathways.

This study had several important limitations. We could not analyze the hepatocyte differentiation marker HNF4α [50] or further investigate the expression patterns of key biliary markers [42]. Our decision to concentrate on both the early and late periods, rather than focusing exclusively on the early phase, could be considered a limitation. Lastly, while we did not perform knock-out or knock-in studies, our results may provide a valuable foundation to guide such investigations in the future.

## 5. Conclusions

In conclusion, in the ADSC-treated group, biliary damage was reduced, liver fibrosis was prevented, and liver functions were restored via changes in Hes1 protein and gene expression. Future research using techniques such as laser microdissection could help to evaluate gene expression changes, specifically within the portal triads and the periportal area versus other hepatocytes distributed throughout the lobule. This approach would provide a clearer understanding of the definitive effects of ADSC on hepatic and biliary regeneration.

## Data Availability

The raw data supporting the conclusions of this article will be made available by the authors upon request.

## Supplementary Materials

The following supporting information can be downloaded at: https://www.mdpi.com/article/10.3390/biomedicines14030657/s1, Table S1: AST, ALT, ALP, GGT, Total Bilirubin, and Direct Bilirubin Levels Were Evaluated Among the Study Groups. All Results Were Expressed as Median ± IQR, and the Statistical Significance Level Was *p* < 0.05 for the Analysis. * versus Sham group; Table S2: Pairwise Comparisons of Study Groups.
