# Supplementary material for: Adipose-Derived Stem Cell Treatment Induces Early-Term Hes1 Upregulation in a Sox9- and Notch1-Independent Manner in a Rat Model of Bile Duct Ligation
Source: Biomedicines. 2026 Mar 13;14(3):657. doi: 10.3390/biomedicines14030657 (PMC13024265; doi:10.3390/biomedicines14030657)
Supplement: Supplementary file 1 [file biomedicines-14-00657-s001.zip › biomedicines-4117769-supplementary.pdf]

Supplementary Table S1. AST, ALT, ALP, GGT, total bilirubin, and direct bilirubin levels were evaluated among the study groups. All results were expressed as median ± IQR, and the statistical significance level was $p < 0.05$ for the analysis. * versus Sham group.

	AST	ALT	ALP	GGT	TBIL	DBIL
**Sham**	118.5± 11,74	52.5± 4,5	98± 17,17	5± 1,4	0.15± 0,054	0.055± 0,005
**Control POD5**	1519.5± 605,99 *	400.5± 13,43 *	296± 135,76	23.5± 17,67	20.5± 8,48 *	12.81± 3,14 *
**ADSC POD5**	278.5± 147,21 *	250.5± 2,12 *	278.5± 154,85	13± 2,828	13.95± 0,35	10.71± 1,835 *
**Control POD15**	1287.5± 139,3 *	215± 43,84 *	261.5± 21,92 *	25.5± 6,36 *	14.35± 0,636 *	10.255± 0,162 *
**ADSC POD15**	319± 196,4	96.5± 19,13	170.5± 82,37	3.5± 6,39	1.05± 4,42	0.605± 3,76

Supplementary Table S2. Pairwise comparisons of study groups

Comparisons	AST (p values)	ALT (p values)	GGT (p values)	ALP (p value)	Total Bilirubin (p values)	Direct Bilirubin (p values)
**Sham vs ADSC POD15**	0.070	0.124	0.318	0.065	0.089	0.030
**Sham vs Control POD15**	0.000	0.000	0.001	0.009	0.002	0.002
**Sham vs Control POD5**	0.007	0.001	0.074	0.188	0.008	0.003
**Sham vs ADSC POD5**	0.013	0.003	0.125	0.034	0.011	0.008
**ADSC POD15 vs Control POD15**	0.345	0.260	0.691	1.000	1.000	1.000
**ADSC POD15 vs Control POD5**	0.950	0.257	1.000	1.000	0.909	0.875
**ADSC POD15 vs ADSC POD5**	0.794	0.259	1.000	1.000	0.666	0.842
**Control POD15 vs Control POD5**	1.000	1.000	1.000	1.000	1.000	1.000
**Control POD15 versus ADSC POD5**	1.000	1.000	1.000	1.000	1.000	1.000
**Control POD5 vs ADSC POD5**	1.000	1.000	1.000	1.000	1.000	1.000
